# Accessing Livestock Resources in Ensembl

**DOI:** 10.3389/fgene.2021.650228

**Published:** 2021-04-28

**Authors:** Fergal J. Martin, Astrid Gall, Michal Szpak, Paul Flicek

**Affiliations:** European Molecular Biology Laboratory, European Bioinformatics Institute, Wellcome Genome Campus, Cambridge, United Kingdom

**Keywords:** Ensembl, genome browser, annotation, tutorial, livestock, farmed animals, Ensembl VEP, REST API

## Abstract

Genome assembly is cheaper, more accurate and more automated than it has ever been. This is due to a combination of more cost-efficient chemistries, new sequencing technologies and better algorithms. The livestock community has been at the forefront of this new wave of genome assembly, generating some of the highest quality vertebrate genome sequences. Ensembl’s goal is to add functional and comparative annotation to these genomes, through our gene annotation, genomic alignments, gene trees, regulatory, and variation data. We run computationally complex analyses in a high throughput and consistent manner to help accelerate downstream science. Our livestock resources are continuously growing in both breadth and depth. We annotate reference genome assemblies for newly sequenced species and regularly update annotation for existing genomes. We are the only major resource to support the annotation of breeds and other non-reference assemblies. We currently provide resources for 13 pig breeds, maternal and paternal haplotypes for hybrid cattle and various other non-reference or wild type assemblies for livestock species. Here, we describe the livestock data present in Ensembl and provide protocols for how to view data in our genome browser, download via it our FTP site, manipulate it via our tools and interact with it programmatically via our REST API.

## Introduction

Efficient management of livestock resources is key to global food security. Livestock production represents the largest land use sector worldwide and employs almost a billion people globally (Hurst et al., 2005; Abu Hatab et al., 2019). Livestock production is critical to developing countries, as it acts both as a major source of income and a means to escape poverty (Otte and Upton, 2005) and as a backup food source in the case of crop failures (Kabubo-Mariara, 2009; Rota and Sperandini, 2009). As the world’s population continues to grow, so too does the demand for livestock source foods (LSFs). LSFs and other animal products account for approximately one-third of human protein consumption (Popp et al., 2010). The average per capita meat consumption is projected to grow from 34 kg in 2015 to 49 kg in 2050 (Yawson et al., 2017). At the same time, there is increasing competition for the use of key resources such as land and water and a need to move to less carbon intensive LSF production, especially in the face of climate change (Thornton, 2010; Yawson et al., 2017). Over the past decade, genomics has emerged as a key tool in the effort to create more efficient LSF production, particularly the use of genomic selection to improve breeding programs (Hayes et al., 2009; Christensen et al., 2012; Cleveland and Hickey, 2013).

The livestock community has been at the forefront of genomics in terms of generating high quality genome assemblies and accompanying transcriptomic data, which are key to generating detailed genome annotations and exploring genomic variation among populations. Species such as pig, chicken, cow, horse, sheep, goat, salmon and herring all have chromosome-level genome assemblies, suitable for detailed annotation and downstream analyses (Jiang et al., 2014; Lien et al., 2016; Bickhart et al., 2017; Warren et al., 2017; Kalbfleisch et al., 2019; Pettersson et al., 2019; Warr et al., 2020).

In addition to reference genomes, an increasing number of alternative genome assemblies are available for analysis. Several breed-specific genomes have been sequenced and assembled including a large number of pig breeds (Fang et al., 2012; Li et al., 2017; Warr et al., 2020), black Bengal goat (Siddiki et al., 2019), Korean chicken (Sohn et al., 2018), and three strains of common carp (Xu et al., 2019). Livestock species are often at the cutting edge of genome assembly, exemplified by the recent trio binning approach (Koren et al., 2018) used to fully separate the maternal and paternal genomes from a *Bos indicus* × *Bos taurus* hybrid individual (Low et al., 2020).

With this wealth of high-quality genome sequences, it is crucial that the resulting genome annotation is carried out and presented in a clear and consistent manner. Ensembl is a genomics resource built to provide genome annotation and enable consistent interpretation of genomic variation both within and across species (Howe et al., 2021). The mission of Ensembl is to accelerate downstream science by providing pre-computed analyses, powerful genome interpretation tools and numerous ways of interacting with data through our extensive infrastructure. The data include genome sequences, gene annotation (Aken et al., 2016), comparative analyses (Herrero et al., 2016), variation (Hunt et al., 2018), and regulatory data (Zerbino et al., 2015). Tools such as the Ensembl Variant Effect Predictor (VEP; McLaren et al., 2016) and BLAST/BLAT services allow further interrogation of both the genome sequences and their annotations. Numerous ways are available to interact with the data including our genome browser, FTP site, REST APIs and BioMart querying tool.

In this article we present protocols for interacting with livestock data in Ensembl. We will examine several different livestock species from a variety of perspectives. These include investigating a genomic region of the cow, exploring a gene in chicken, viewing comparative data across pig breeds and annotating variants in goat. For more large-scale analyses, we provide examples of how to programmatically access the data and download the associated annotation files. In summary, readers will get a thorough understanding of the livestock data held in Ensembl and how to work with it.

## Materials

Computer and Internet Connection.

An Internet browser: recent versions of Firefox, Chrome, Safari, and Microsoft Edge are supported.

For working with the REST API, examples are presented in Python 3.

## Methods

The following protocols use Ensembl release 101 (August 2020)^1^. There may be updates to interfaces or data if a more recent release is used.

### Exploring Genes and Genomes

The most fundamental data in Ensembl are the genome sequences and the gene annotation for each species. In this section we will look initially at how to view and explore a region of the cow genome and then examine a gene in the chicken genome. This will form the basis for later explorations of comparative and variation data, which build on data held in the genes and genomes.

#### Browsing a Genome

Much of the annotation in Ensembl corresponds to an underlying genomic region. Becoming familiar with how to browse these regions is key to understanding the annotation available. The following protocol describes how to examine a region of the cow reference genome.

**FIGURE 1:**
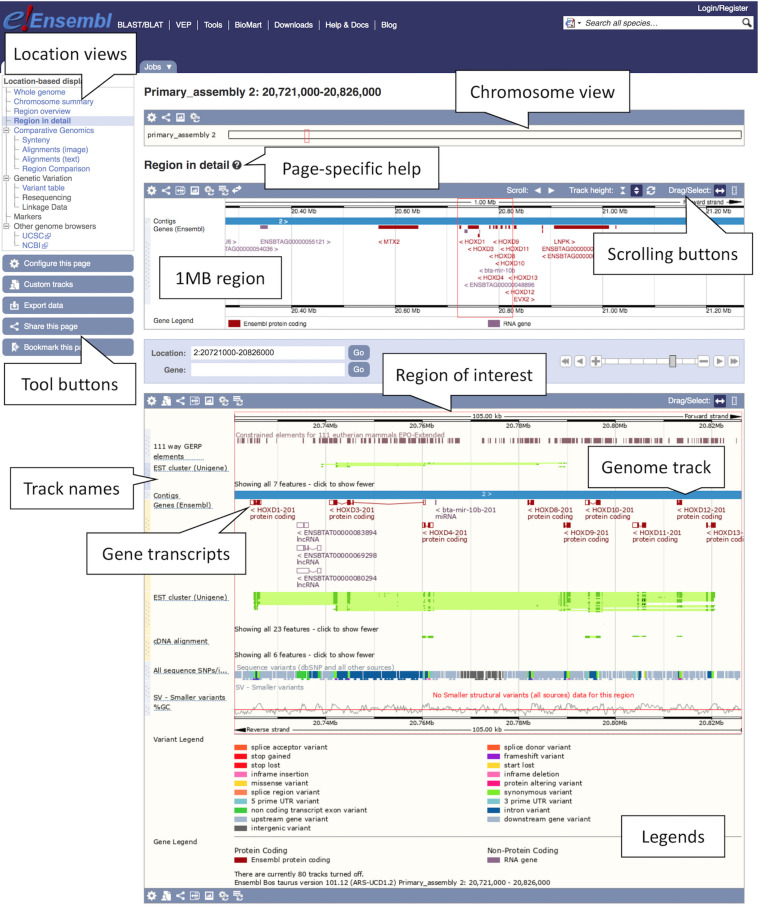
Location view of the *HOXD* gene cluster in cow. In the figure, various tracks are displayed including the main gene track, SNP data, constrained elements and a GC content. Most tracks are disabled by default, tracks such as the tissue-specific short read alignments can be toggled on and off via the “Configure this page” option in the left-hand menu.

1. Getting started: The Ensembl genome browser can be searched using a variety of terms including gene names, genomic coordinates, variant IDs or phenotypes. Go to the Ensembl’s homepage, www.ensembl.org, and locate two search boxes: one in the upper right corner, and another in the middle of the page. Both the main and the corner search box can be used to search all species. Additionally, you can also refine the main search by choosing species of interest from a drop-down list.
2. Finding a region: Type “cow 2:20721000-20826000” into either box and press the return key. A Location tab will open with a “Region in detail” view displaying the region of interest spanning the *HOXD* gene cluster involved in limb development (see Figure 1). You will find available location displays in the left-hand side menu with blue tool buttons below. The “Region in detail” page has three images, each more detailed than the last: (1) the chromosome view at the top, (2) the 1MB region around the region of interest in the middle, (3) the region of interest corresponding to the specified genomic coordinates at the bottom. The region of interest is indicated by a red box in all three images.

3. Getting help: To get page specific help, click on the question mark (?) button next to the “Region in detail” heading. A pop-up help window will open with instructions on how to navigate this page. You will find a description of the page with screenshots and a tutorial video, as well as links to FAQ, glossary and the Ensembl helpdesk.
4. Navigating a region: There are several ways of navigating a genomic region. By clicking over the region and dragging the cursor, you can draw a box in all three images, which opens a pop-up menu with options to “Mark region” and “Jump to region.” You can also scroll along the genome by using the “Scroll” arrow buttons in the middle image or by changing the mouse click mode to “Drag” (double headed arrow icon). The zoom scrollbar enables zooming in and out. Scroll along the genome in the middle image to change the current genomic location. As you scroll, the image below greys out and two blue buttons appear with options to “Update this image” or “Reset scrollable image.”
5. Customising the view: The data in this view is organised in tracks plotted along the genome. You will find separate tracks for different data types such as genes, SNPs, structural variants or contigs representing the genome assembly. Click on the “Configure this page” button on the left to add more data to this view. A pop-up window with a menu listing all currently active tracks will open. You can find a list of all available tracks organised in different categories on the left with a search box above (with the text “Find a track”). Search for “Proteins from UniProtKB” and turn it on as “Labels.” Click on the tick at the top left of the pop-up window or anywhere outside to save and close. You will find the protein track added to the view.
6. Exporting data: Click on the blue “Export data” button on the left to download data for this region.

A data export window will open with different output format options, including FASTA for sequences and various feature formats such as BED, CSV, TSV, GTF, and GFF3.

#### Exploring a Gene

Gene annotation is one of the most commonly used annotation types in Ensembl. It is a composite data type, representing underlying transcripts, exons, and protein products. In this section, we describe how to find and export information about the *SOX5* gene in the chicken genome.

**FIGURE 2:**
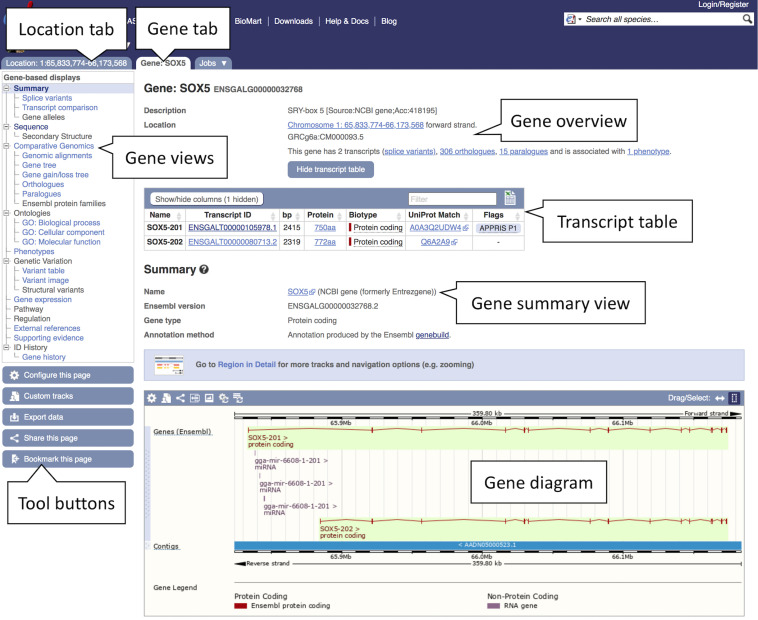
Gene view of *SOX5*. The gene view tab shows a variety of information about the gene including details on the location, transcripts, orthologues, and paralogues. The main gene track is shown in the bottom panel with red blocks representing the exons and connecting red lines representing the introns. In this example a secondary track for liver transcriptomic data can be seen in blue. This and other tissue tracks are available via the “Configure this page” option in the left-hand menu.

1. Getting started: To search for a chicken gene, select “Chicken” from the species selector drop down list above the main search box on the Ensembl homepage, type the gene name, “*SOX5*,” into the search box and click the “GO” button. A list of search results restricted to chicken and matching “*SOX5*” will be generated with the “*SOX5* (Chicken Gene)” at the top.
2. Studying a gene: Click on the “*SOX5* (Chicken Gene)” link to open the Gene tab.
  The Gene tab landing page contains summary information on *SOX5* including its Ensembl stable ID (ENSGALG00000032768), gene description, genomic position, and strand information, as well as the number of transcripts and an option to show them in the tabular format transcript table (see Figure 2). A graphical representation of all transcripts can be found at the bottom of the page. A number of gene-related displays providing additional data can be found in the left-hand menu. The gene overview at the top of this page is visible across all subsequent views.

3. Exploring a gene sequence: Click the “Sequence” display in the left-hand side menu.
  A sequence view of *SOX5* and its flanking region will be displayed in FASTA format with all exonic sequences in this region highlighted in peach colour. Exons of *SOX5* will be indicated by brown bold lettering on top of the peach highlight. You can manipulate this view and change display options by clicking the blue “Configure this page” button on the left. It will allow you to customise the length of the flanking sequence and show genetic variants in the sequence.
4. Downloading a gene sequence: The gene sequence can be downloaded by clicking the “Download sequence” button in the current “Sequence” view or by clicking the blue “Export data” button on the left in any other view. This will open a pop-up window with customisation options that allow to choose different sequence types, the length of the flanking region and the file format (FASTA or RTF).
5. Studying gene ontology (GO): You will find three GO categories under “Ontologies” in the left-hand menu: “GO: Cellular component,” “GO: Molecular function” and “GO: Biological process.” Browse all three views to learn about the gene function. GO terms describe the protein function using standardised vocabulary:
  (a) “GO: Biological process”: what does it do? Example: “positive regulation of chondrocyte differentiation.”
  (b) “GO: Molecular function”: how does it do it? Example: “DNA binding.”
  (c) “GO: Biological process”: where is it located? Example: “nucleus.”
  Each of the above GO categories lists terms associated with transcripts of the *SOX5* gene. The data are organised in a tabular format containing the GO term accession number, the corresponding description, evidence and annotation source, along with the associated transcript stable IDs. On exploring all three views, it emerges that *SOX5* gene encodes a nuclear transcription factor involved in the regulation of chondrogenesis.
6. Exploring external resources: Click “External references” in the left-hand side menu.
  Links to external databases such as Expression Atlas, NCBI, and WikiGene, as well as related RefSeq and UniProtKB/TrEMBL accession numbers can be found here.
7. Studying a transcript: Click the “Show transcript table” button at the top of the page and go to the Transcript tab by clicking the transcript stable ID link “ENSGALT00000105978.1” in the table. The transcript table is visible in any gene and transcript view. It lists all transcripts of the gene of interest, their corresponding name, stable ID, length, biotype and transcript flags indicating transcript quality. You can hover the cursor over the flags to find out more information. In this case the chosen transcript named “*SOX5*-201” has a flag “APPRIS P1.” This means that it is predicted by the APPRIS database (Rodriguez et al., 2018) to be the most functionally important transcript of this gene based on protein structure, functional features and information from cross-species conservation. Similar to the Gene tab, the Transcript tab is also composed of several displays introducing different data types including “Sequence” views and “Protein Information.”
8. Exploring exon sequences: Click “Exons” under “Sequence” in the left-hand side menu to see exon information in tabular format. The Exons view displays a table listing exons, their order in the transcript, genomic position, start and end phase, length, and sequence. Translated sequence is marked in blue, untranslated region (UTR) in orange, flanking sequence in green and introns in grey. This transcript has a 5′ UTR spanning the entire first exon and the beginning of the second exon. “Configure this page” will allow you to customise this view.

### Comparing Genes and Genomes

Ensembl has a powerful comparative genomes infrastructure to deliver information about how genes and genomes relate to one another across our supported species. Here, we describe assessing comparative data, focusing on gene trees and whole genome alignments.

#### Examining Genes Trees and Orthologous Genes in Pig

A common way to assess the reliability of annotation of a gene, both in terms of structure and function, is to examine orthologous genes in other species. Genes that are present across a broad range of species, with high sequence similarity and within syntenic regions, are likely to have equivalent functions. Understanding the level of conservation of a gene across species can assist with downstream inference and analysis. Here, we will examine a highly conserved gene, *FILIP1*, in pig and explore the associated gene tree as well as data for *FILIP1* orthologues in other species.

**FIGURE 3:**
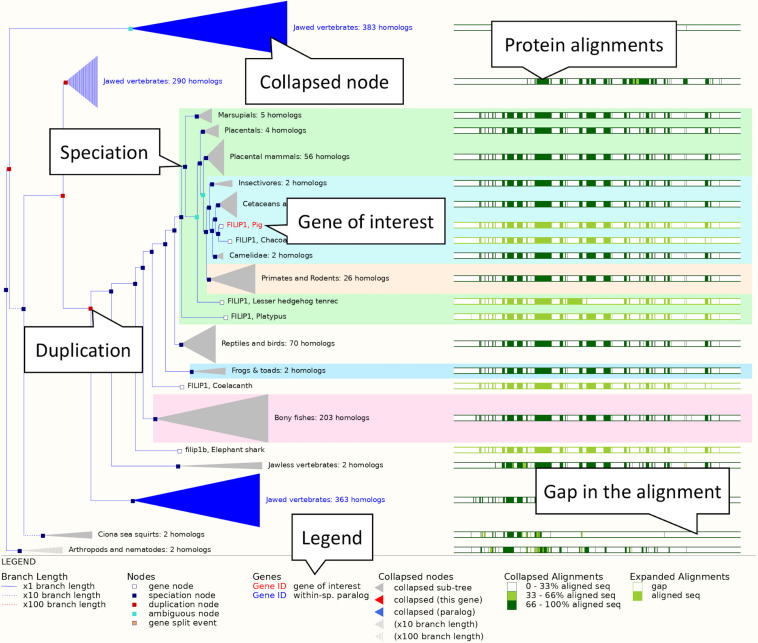
The *FILIP1* gene tree. The pig FILIP1 gene is highlighted in red. Various other clades are collapsed into funnels to improve usability of the tree. Subtrees can be expanded and collapsed by clicking on the corresponding node (represented by a square on the tree) and selecting the appropriate option from the menu. The right-hand side shows a visual representation of alignment conservation of the corresponding protein sequences across the tree.

1. Getting started: Select “Pig” from the species selector drop down list above the main search box on the Ensembl homepage, type “*FILIP1*” into the search box and hit the “GO” button.
  A list of search results across all breeds will be generated. In the left-hand menu you will see options for filtering the search results. For species like pig, where there are alternative breeds/strains available, we have a defined reference. In general, for livestock species, the reference chosen is based on feedback from the community. In the case of pig, the current reference chosen by the community is the Sscrofa11.1 assembly of the Duroc breed. Try restricting the results to the *FILIP1* gene in the reference breed. First, in the left-hand menu under “Restrict breeds to” click on “Pig reference.” Next, under “Restrict category to” click on “Gene.” The initial results are now filtered to just genes in the reference breed matching the name “*FILIP1.*” You will now have two results representing the *FILIP1* and *FILIP1L* genes. Click on the “*FILIP1* (Pig Gene, Breed: reference)” link on top to go to the Gene tab. This will present the gene view for the *FILIP1* gene in the reference pig genome.
2. Exploring a gene tree: In the left-hand side menu, click on the “Gene tree” display. An image showing a phylogenetic tree will be loaded. The current gene, marked in red, is shown in the context of homologous genes found across various clades including primates, rodents, birds, reptiles and even non-vertebrates such as *Caenorhabditis elegans* and *Drosophila melanogaster* (see Figure 3). Grey funnels indicate collapsed nodes, which can be expanded by clicking on them and selecting “expand this sub-tree” from the pop-up menu. A graphical representation of the protein alignment used to calculate this tree can be found on the right. Green colour indicates aligned sequences, while alignment gaps are shown in white. A consensus alignment is displayed for collapsed nodes, with two shades of green corresponding to the proportion of aligned collapsed sequences. Black vertical lines mark exon-intron boundaries.

3. Retrieving orthologues: Click “Orthologues” in the menu on the left. This will load the orthology table.
4. The table lists *FILIP1* orthologues found across a large range of species. To make it easier to examine the data click on the show details box beside “Laurasiatheria” in the species set list. This will restrict the data in the orthologue table below to just laurasiatherian species.
5. Examine the column headings in the orthologue table to see the types of information available in the table. Some columns have extra information that displays when the mouse cursor is left over them.
6. Scroll down to the orthologue for horse. The sequence identity of the pig *FILIP1* gene with the horse *FILIP1* gene is high. The query and target ID percentages, which represent how much of the pig *FILIP1* sequence matches the horse *FILIP1* sequence and vice-versa are both over 94%, indicating strong conservation. The gene order conservation score, which represents conserved orthology between the two nearest 5′ and 3′ genes flanking *FILIP1*, and whole genome alignment coverage are both 100. This implies strong gene order conservation and a high coverage pairwise alignment of the broader underlying genomic regions. As a result, the orthologue is listed as high confidence, as indicated by a “Yes” in the “High Confidence” column.
7. Exploring a protein alignment: Click on the “View Sequence Alignments” link to open a pop-up menu with options to view protein and cDNA alignment. Click “View Protein Alignment.” An alignment of the gene of interest and its orthologue will be displayed in CLUSTAL W format. Click on the question mark button (?) next to the “Orthologue alignment” heading for more information on the conservation codes.

#### Viewing a Whole Genome Alignment of Pig Breeds

Ensembl provides a large number of pairwise and multiple whole genome alignments. Every species has a pairwise alignment against a reference species for its clade. The reference species for mammals, birds and fish are human, chicken, and zebrafish, respectively. For some species, additional pairwise alignments are generated. For example, rodent genomes are aligned against the mouse reference, while the pig reference has a pairwise alignment to the USMARC pig assembly. In addition to the pairwise alignments, various multiple whole genome alignments are available, including the 57 mammals and the 95 amniota vertebrates alignments.

Here, we will look at a multiple alignment generated for 13 pig breeds and three outgroup species: cow, horse, and sheep (texel). Using the *COL12A1* gene, we will see that this region is generally well conserved across the alignment, however the gene is truncated in the Tibetan breed. We will examine the alignment for potential explanations for this truncation.

**FIGURE 4:**
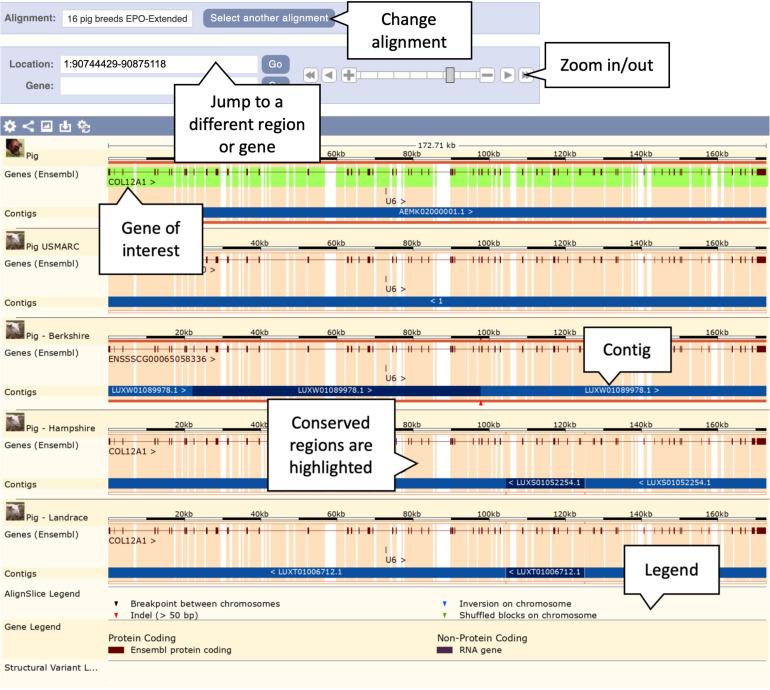
Genomic alignment of pig breeds centred on the *COL12A* gene. This figure shows only the first five alignments including the reference pig at the top, the complete alignment contains all 13 pig breeds along with cow, horse, and sheep as outgroups. The pale orange highlighting in the background represents conservation. Exonic regions are naturally more conserved across the alignment.

1. Getting started: From the Ensembl frontpage type “pig 1: 90744429-90875118” into the search box and click the “GO” button. This will bring you directly to the genomic location of the *COL12A1* gene in the pig reference genome.
2. Using the left-hand side menu, click on “Alignments (text)” under “Comparative Genomics” to access available whole genome alignments for the pig reference genome.
3. Click on the “Select an alignment” button to see the alignment selector tool.
4. Choose “16 pig breeds EPO-Extended” from the multiple alignment category. This provides a multiple whole genome alignment of all pig genomes in Ensembl and the three outgroup species generated from the Enredo, Pecan, and Ortheus pipeline (Paten et al., 2008). A graphical representation of an expanded phylogenetic tree and corresponding section of the whole genome alignment will load, followed by a list of the aligned regions and a preview of the sequence alignment.
5. Examine the list of aligned regions. The alignment from the reference to the USMARC assembly is represented by a single contiguous alignment block in the USMARC assembly, reflecting the high-quality and contiguity of the assembly. This is also true for the aligned regions of the three outgroup assemblies (cow, sheep, and horse). For other pig breeds, there are multiple alignment blocks due to the lower contiguity and completeness of the assemblies. In particular, the Tibetan and Wuzhishan alignments are fragmented across multiple genomic regions, implying the region is not correctly reconstructed in these assemblies.
6. Click on the “View an image of this alignment” link, located directly above the list of regions. This will load a more detailed view of the *COL12A1* gene structure across the aligned regions, as shown in Figure 4.

7. Examine the gene structure across the breeds. Note that the intron/exon structures are mostly well conserved across breeds. Scroll down to the Tibetan copy of the *COL12A1* gene. Note that the gene is heavily truncated in comparison to the other breeds. The alternating light and dark blue bands represent the boundaries of different alignment blocks and are labelled with the region of the genome each block comes from. For the Tibetan breed the annotated section of the gene lies on the AORO02005858.1 scaffold. The remainder of the alignment blocks are on the AORO02052718.1 scaffold. This provides strong evidence that parts or all of these two scaffolds should have been joined in the Tibetan assembly. As a result of them not being joined the *COL12A1* gene is truncated at the end of the AORO02005858.1 scaffold, where the majority of the gene resides.

#### Viewing a Synteny Map of Pig Chromosome 6 to Human

Whole genome alignments can also be used to generate synteny maps between chromosomes of different species. These maps show genomic regions in which genes occur in the same order in two species. This view gives insight into how chromosomes have diverged between two species and any two species with pairwise whole genome alignments can be compared in this way. Here, we will describe viewing a synteny map between pig chromosome 6 and the corresponding regions in the human genome.

**FIGURE 5:**
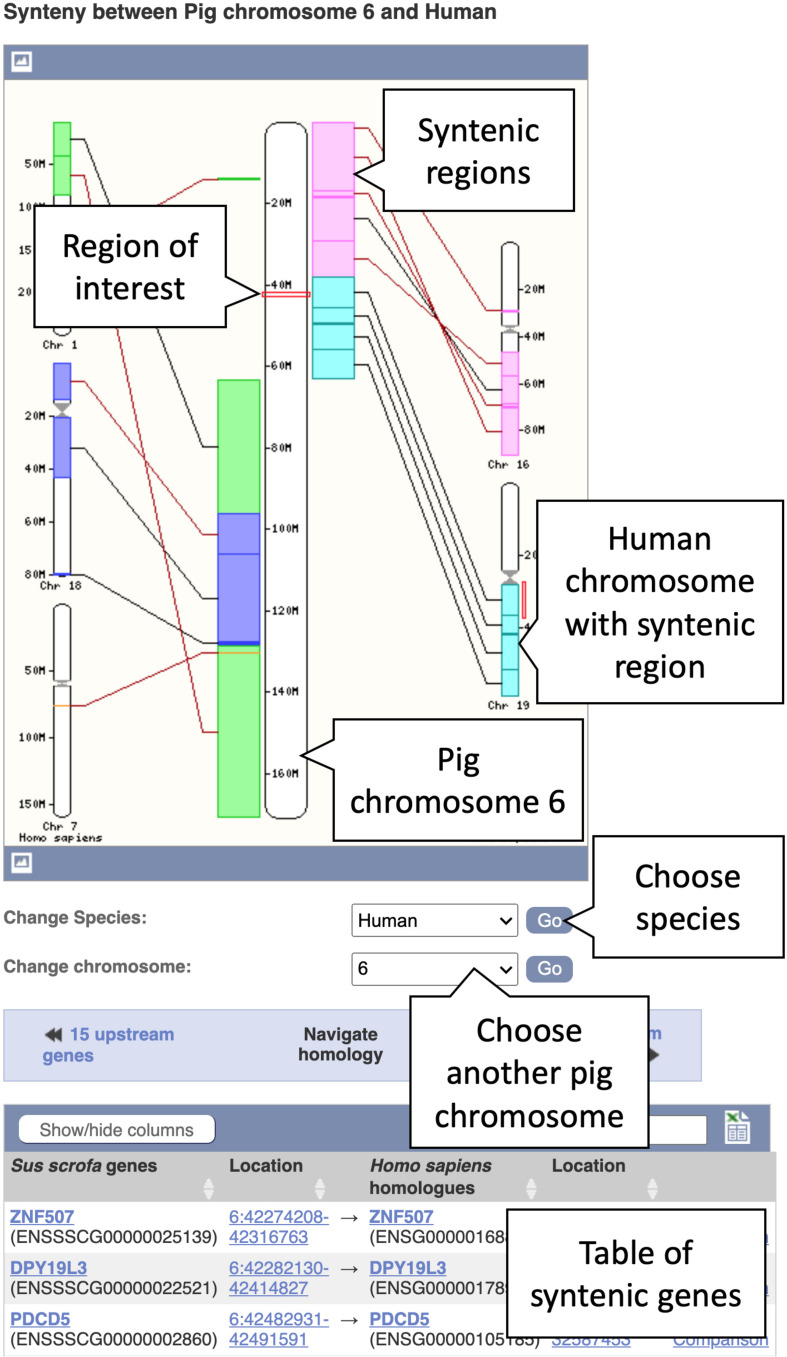
A synteny map from pig chromosome 6 to human. Regions on pig chromosome 6 (located in the centre) are syntenic to multiple regions on human chromosomes 1, 7, 16, 18, and 19 (shown on the left- and right-hand sides). Syntenic blocks are coloured according to the chromosome numbers for human. The blocks are connected by lines, whereby black lines connect blocks with the same orientation and brown lines indicate blocks with the opposite orientation.

1. Getting started: From the Ensembl homepage click the dropdown box under the “All genomes” heading (the box will have “–Select a species–” by default).
2. This will produce a list of species grouped under headings, including major clades such as primates and rodents. Scroll down to “Laurasiatheria” and click on “Pig” to go to the species page for the reference pig genome
3. From the species page select “View karyotype.” This will give the karyotype view of all chromosomes in the reference pig. Click anywhere within chromosome 6 on the image of the karyotype to see a pop-up window.
4. Select “Jump to region overview” in the pop-up window. This will bring you directly to the Location tab of the corresponding region on chromosome 6.
5. In the Location tab, select “Synteny” under the “Comparative Genomics” section in the left-hand side menu to bring up the synteny view for pig chromosome 6. You will now see an image of a synteny map between chromosome 6 in pig and the various chromosomes in human that it maps to (see Figure 5). The region you selected and the corresponding location in human are indicated by red boxes. Syntenic blocks are shown in different colours and connected by lines. You can change the chromosome or select species other than human, where a pairwise whole genome alignment is present, using the drop-down on the right.

### Examining Genetic Variation and Annotating Variants

Several livestock species including chicken, goat, pig, and salmon have extensive variation data. Ensembl provides extensive views via the bowser and also analysis of variant data via the Ensembl Variant Predictor (VEP). In this section we will show how to browse and analyse variant data in the goat genome.

#### Exploring Variation Data

Variation data can be accessed in a number of ways through the browser including by selecting it in the configuration menu and by navigating to the “Genetic variation” section in the left-hand menu on the Location, Gene, and Transcript tab. There are also example entry points on each species page and support for searching variant identifiers from dbSNP and other databases. Here, we will examine different aspects of variation data including population frequencies, phylogenetic context, and consequences.

**FIGURE 6:**
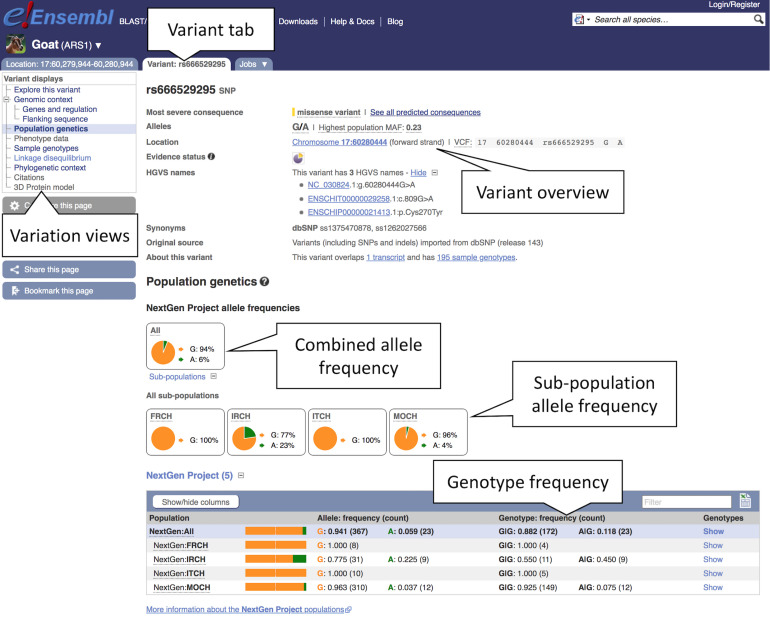
Exploring variation data in goat. Here, we see the view of population genetics data within the variant tab. Pie charts for the overall allele frequencies along with frequencies for subpopulations are provided based off the NextGen Project data.

1. Getting started: Type “rs666529295” into the main or the upper right corner search box to search all species and hit return. The search result page will return two hits with “rs666529295 (Goat Variant, Breed: reference)” at the top, click on this link.
2. Studying a variant: You will be taken to the variant summary page containing variant overview information such as the most severe consequence, variant alleles as reference/alternative (here: “G/A”), highest population minor allele frequency (MAF), genomic location and strand.
3. Exploring variant allele frequencies: Click the “Population genetics” icon or the link in the left-hand menu to display allele frequencies from the NextGen Project (Alberto et al., 2018). The pie chart shows the allele frequency across all sequenced goat populations (see Figure 6). Click the “ + ” next to “Sub-populations” to reveal the allele frequencies in the included sub-populations.

4. Studying the phylogenetic context: Click “Phylogenetic context” to see the conservation of this variant and its flanking region across different taxa. Click “Select an alignment,” then “Multiple.” This should show a list of available multiple species alignments. Select “95 amniota vertebrates Mercator-Pecan” and click “Apply” to load the alignment. The resulting page displays a multiple alignment of 10 bp around the focus variant. In this case the variant is conserved across all taxa.
5. Transcript consequences: Click “Genes and regulation” under “Genomic context” in the menu to see which genes and transcripts are affected by this variant. In this case, we can see that a transcript in the *EDNRA* gene is affected with a consequence type of “Missense variant.” The location in both the transcript and CDS sequence are shown, along with the codon and amino acid changed. The Sorting Intolerant From Tolerant (SIFT) pathogenicity prediction score (Kumar et al., 2009) is “0,” indicating that the amino acid substitution is predicted to be deleterious to the function of the protein.
6. The Variant table: By clicking the Gene stable ID link “ENSCHIG00000019737,” you can navigate directly to the Gene tab and be taken straight to the “Variant Table” display. This lists all variants in the Ensembl database that fall within the *EDNRA* gene (including a 5 kb flanking region). Data can be downloaded as a CSV file by clicking the Excel icon.
7. Studying a phenotype: From the left-hand menu in the Gene tab, click “Phenotypes” to explore the complete set of phenotypes, diseases and traits associated with the gene. This gene has been associated with “Coat colour, white spotting, *EDNRA*-related” according to OMIA (Lenffer et al., 2006). There are no variants for this gene currently associated directly with phenotypes. Toward the bottom of the page a list of phenotypes for orthologues of the gene is provided to help cross-species phenotypic comparison. Clicking on the link for any of the phenotypes listed on the page will provide a list of other loci present in the species that are associated with the same phenotype.

#### Annotating Variants With the Ensembl VEP

The Ensembl VEP (McLaren et al., 2016) classifies the impact of variants on genes, transcripts, and protein sequences and identifies known variants that match the input variants.

The Ensembl VEP is available as a web interface, a command line tool and through a REST API endpoint. The web interface is suitable for smaller amounts of data, while the command line tools is suitable for large-scale analysis and offers maximum flexibility, including the option to analyse variants for genomes that are not in Ensembl. Here, we’ll look at the use of the point-and-click web interface to analyse six goat variants input in Variant Call Format (VCF).

**FIGURE 7:**
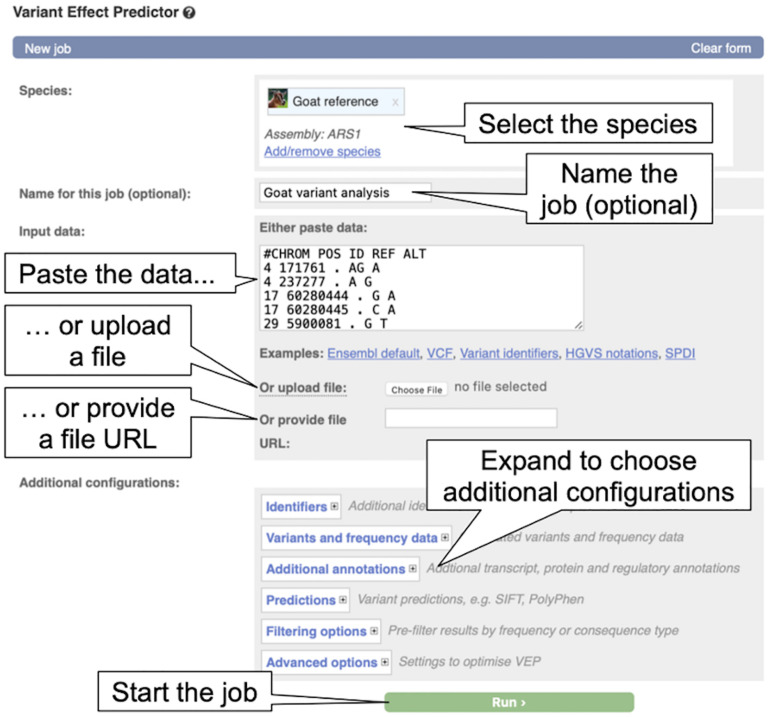
The Variant Effect Predictor input form. The overlayed dialogue boxes provide a breakdown of the steps involved in submitting data to the VEP.

**FIGURE 8:**
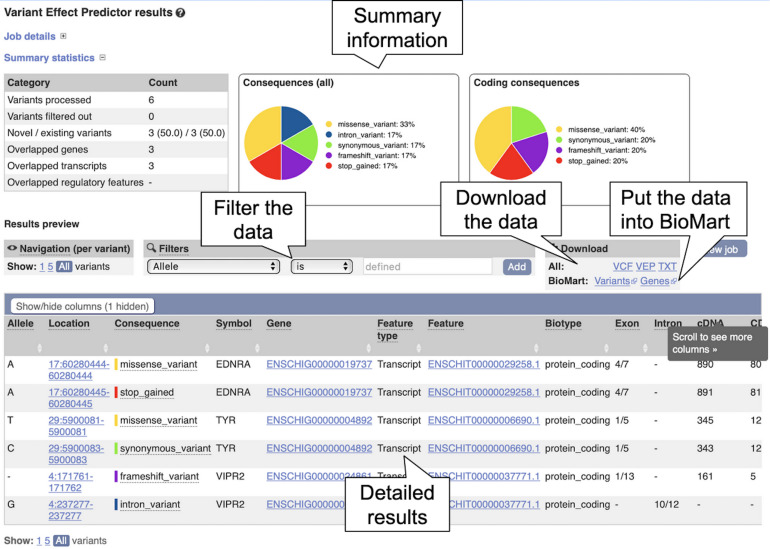
The Ensembl Variant Effect Predictor results page. The summary information at the top shows the consequence information for the uploaded variants. The results table at the bottom shows more detailed information for each uploaded variant including overlapping genes and transcripts.

1. From any page in Ensembl, click on the link to “VEP” at the top of the page.
2. From the VEP page, click on “Launch VEP” in the “Web interface” box to load the VEP input form (see Figure 7).

3. By default, human is selected as in the species list, click on the “X” beside “Homo_sapiens” to remove human from the species list. Now click “Add/remove species” to load the species selector box. When it pops up, begin to type “goat” and select “Goat reference (Capra_hircus)” and click “Apply.” The reference goat genome should now be selected in the VEP input form.
4. Copy the below variant data into the “Input data” box.
  #CHROM POS ID REF ALT
    4 171761. AG A
    4 237277. A G
    17 60280444. G A
    17 60280445. C A
    29 5900081. G T
    29 5900083. G C
    In this case we will run with default parameters, but the reader is encouraged to take a look at the various options available for configuring the VEP.
5. Click on the “Run” button. The display shows the status of the job. It will say “Queued,” then switch to “Done” when the job has finished. It is possible to save, edit, share or delete a job by using the icons on the right. If multiple jobs are submitted, they will appear in this table.
6. Click on “View Results” once the job is done. At the top of the results page, three sets of summary information are displayed (see Figure 8). The table shows that six variants have been processed, none has been filtered out, three existed already and that the variants overlap three genes and three transcripts. Pie charts show the proportions of total and coding consequences predicted: two missense variants, one intron, frameshift, and synonymous variant each, and one variant that introduces a stop codon. At the bottom of the page, a table with detailed results is displayed. It includes the alleles used for the predictions, the location of the variants, their consequences and other useful information.

### Accessing and Downloading Large Data Sets

Up until this point we have focused on viewing data relating to individual loci in the context of the genome browser. To enable analysis of data over larger regions we provide a number of different methods for bulk data access. In this section, we will focus on three different methods: creating queries in our BioMart data exporter, programmatic access via our REST API and downloading files via our FTP site.

#### BioMart: Retrieving NCBI Gene IDs, GO Terms, and cDNA Sequences of Sheep Genes

BioMart (Kinsella et al., 2011) enables the creation of complex queries on data in Ensembl. The results can then be exported in different formats depending on the type of data queries. The underlying databases can also be accessed programmatically using R with the Bioconductor package biomaRt (Durinck et al., 2005). Data retrieval using BioMart is possible for medium to large datasets with hundreds of entries, but it is not suitable for whole genome-scale data.

The following BioMart queries first generate a CSV file with NCBI gene IDs and GO terms for the sheep genes *ESPN*, *USH1C*, *CISD2*, *THRB*, *GIPC3*, and *BRCA2* (query #1 below) and the get their cDNA sequences in FASTA format (query #2 below). In all BioMart queries a dataset must be selected, filters set (input – here the six gene names) and attributes (desired output) defined before the results can be exported.

**FIGURE 9:**
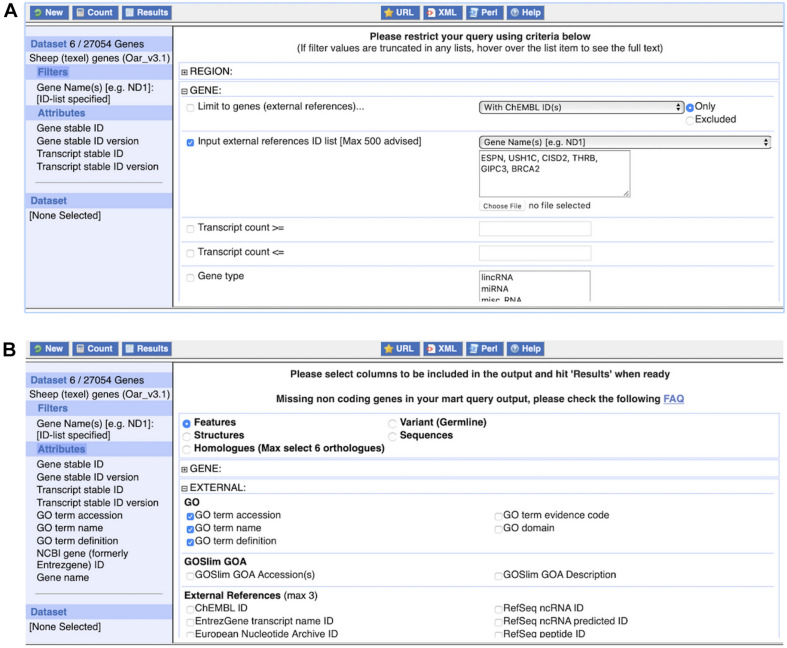
The BioMart web interface. Panel **(A)** shows the application of one commonly used filter, an external references identifier list, here consisting of six gene names. The dataset size of six genes is displayed after clicking on “Count” at the top left. Panel **(B)** shows the selection of attributes, here “GO term accession,” “GO term name,” and “GO term definition.” The complete list of attributes, i.e., what will be included in the results table, is shown on the left.

(1) Query 1: Click on “BioMart” at the top of any Ensembl page to load BioMart. You should see an interface similar to Figure 9.

1. Under “Dataset,” Choose the “Ensembl Genes” database and the “Sheep (texel) genes (Oar_v3.1)” dataset from the respective drop-down menu.
2. Click on “Filters” in the left panel. Expand the “GENE” section by clicking on the “ + ” box. Select “Input external references ID list” and paste “*ESPN, USH1C, CISD2, THRB, GIPC3*, *BRCA2*” in the text box. Select “Gene Name(s)” from the drop-down menu.
3. Click on “Count” to check the Filters. This shows six genes.
4. Click on “Attributes” in the left panel. Expand the “EXTERNAL” section by clicking on the “ + ” box. Select “GO term accession,” “GO term name,” “GO term definition” and “NCBI gene (formerly Entrezgene) ID.” Then expand the “GENE” section by clicking on the “ + ” box. The Ensembl “Gene stable ID” and “Transcript stable ID” are pre-selected. In addition, select “Gene name” to include the input in the CSV file.
5. Click “Results.” Select Export all results to “File” and “CSV” from the drop-down menus. Click on the “Go” button to export the file.

(2) Query 2: Click on “Attributes” again. Do not change Dataset and Filters.
  1. Select the “Sequences” attributes page at the top. Expand the “SEQUENCES” section by clicking on the “+” box. Select “cDNA sequences.” Then expand the “HEADER INFORMATION” section by clicking on the “+” box. As before, Ensembl “Gene stable ID” and “Transcript stable ID” are pre-selected. Select “Gene name” to include the input in the FASTA file.
  2. Click “Results.” Select Export all results to “File” and “FASTA” from the drop-down menus. Click on the “Go” button to export the file.

#### REST API: Retrieving Homologues of a Horse Gene

The Ensembl REST API (Yates et al., 2015) is available at rest.ensembl.org and its user guide, including a Getting Started section, at http://github.com/Ensembl/ensembl-rest/wiki. Our REST API consists of a variety of endpoints. Endpoints can be considered parts of the API that allow retrieval of particular types of data. The data these endpoints provide includes transcript sequences, meta data, gene trees, variants, and a host of others.

The following Python script uses the GET homology/symbol/:species/:symbol endpoint to retrieve homologues of the horse *BRCA2* gene and print information about them in FASTA format. It uses a helper function to make the request, check for errors and decode the JSON response (the function returns text if the content_type is not JSON). The function can be integrated in any script to simplify these steps.

#!/usr/bin/env python

# Get the necessary Python modules

import requests, sys, json

# Define a helper function

def fetch_endpoint(server, request, content_type):

# Make the request

r = requests.get(server + request, headers = {”Accept”: content_type})

# Get the status of any failed query

if not r.ok:

r.raise_for_status()

sys.exit()

# Decode JSON, if used as content_type. If not, return text.

if content_type = = “application/json”:

return r.json()

else:

return r.text

# Define the gene name

gene = ”BRCA2”

# Define the general URL parameters

server = “http://rest.ensembl.org/”

ext_hom = “homology/symbol/horse/” + gene

con = “application/json”

# Submit the query by calling the helper function

get_hom = fetch_endpoint(server, ext_hom, con)

# Print some information about the homologues

for data in get_hom[“data”]:

for homology in data[“homologies”]:

source_id = homology[“source”][“id”]

source_species = homology[“source”][“species”]

source_seq = homology[“source”][“align_seq”]

target_id = homology[“target”][“id”]

target_seq = homology[“target”][“align_seq”]

target_species = homology[“target”][“species”]

print (“>,” source_id + “ ” + source_species + “\n” + source_seq + “\n>,” target_id + “ ” + target_species +“\n” + target_seq)

#### FTP: Downloading a GTF File for Atlantic Salmon

The Ensembl FTP site at ftp://ftp.ensembl.org/pub/can be accessed using the command line, a script, rsync, a web browser or FTP client. It provides files for all species in several formats, such as FASTA, GTF/GFF3 and VCF for the current and previous releases (going back to release 19). An overview of available data can be found at https://www.ensembl.org/info/data/ftp/index.html.

To download a GTF file with all annotated transcripts for Atlantic salmon using a web browser:

1. Navigate to ftp://ftp.ensembl.org/pub/
2. Click on current_gtf and then on salmo_salar
3. Click on the file Salmo_salar.ICSASG_v2.100.gtf.gz

## Results

Here, we present a summary of some of the key livestock data present in Ensembl, including the genomes, different annotation types and FTP files.

### Livestock Species in Ensembl

Ensembl contains a large variety of livestock species ranging from cow, pig, and chicken to Arabian camel, African ostrich, and Siberian musk deer. In addition, there are some species with accompanying non-domesticated genomes, such as common and wild mallard or domestic and wild yak. Table 1 shows ten of the major livestock species in Ensembl along with some accompanying information about the genome assembly and the Ensembl release which included the most recent update to the associated annotation.

**TABLE 1:** Assembly statistics for ten reference livestock species in Ensembl.

**Species**	**Assembly**	**Accession**	**Contig N50**	**Date**	**Release**
Chicken	GRCg6a	GCA_000002315.5	17655422	2018-03-27	95
Duck	CAU_duck1.0	GCA_002743455.1	88037	2017-11-03	96
Cow	ARS-UCD1.2	GCA_002263795.2	25896116	2018-04-11	98
Goat	ARS1	GCA_001704415.1	26244591	2016-08-24	92
Horse	EquCab3.0	GCA_002863925.1	1502753	2018-01-05	98
Pig	Sscrofa11.1	GCA_000003025.6	48231277	2017-02-07	98
Sheep	Oar_rambouillet_v1.0	GCA_002742125.1	2572683	2017-11-02	101
Herring	Ch_v2.0.2	GCA_900700415.1	1151065	2019-04-16	98
Seabream	fSpaAur1.1	GCA_900880675.1	2862625	2019-07-30	99
Salmon	ICSASG_v2	GCA_000233375.4	36085	2015-06-10	99

*Date refers to the date the assembly was submitted to the public archives. Release is the Ensembl release number in which the annotation for the corresponding assembly was most recently updated.*

Many of the livestock species, particularly the more recently sequenced ones, have high quality genome assemblies based on long read sequencing. Several species including chicken, cow, goat, and pig have genome assemblies with a contig N50 of over 10 Mb. That being said, there is considerable variability in the quality of livestock assemblies in Ensembl as many species were assembled using short read data. We see a range of contig N50 values, from approximately 30 kb to over 48 Mb, representing over a 1,000-fold variation in the level of contiguity of these assemblies.

### Gene and Transcript Annotation Across Livestock Species

**FIGURE 10:**
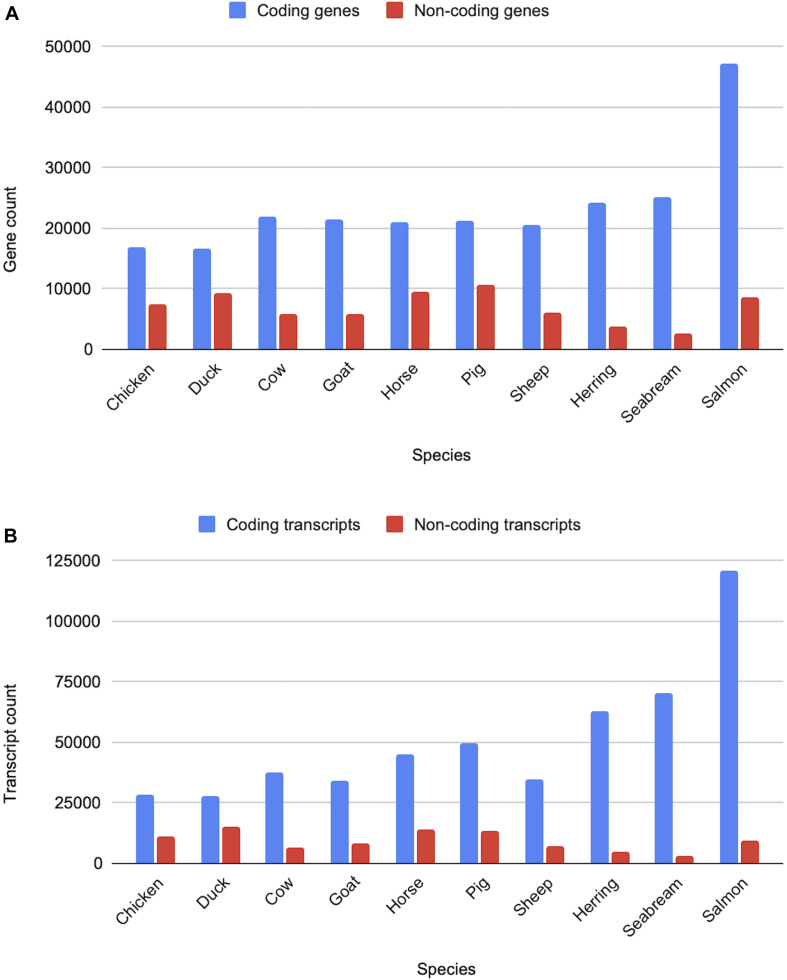
Counts of coding and non-coding genes and transcripts for ten livestock species. Panel **(A)** shows the gene counts, while panel **(B)** shows the transcripts counts for various livestock species from the bird, mammal and fish clades.

All livestock species have gene sets generated via the Ensembl gene annotation system (Aken et al., 2016). The counts of the coding and non-coding genes and transcripts across ten livestock species are shown in Figure 10. Across clades a consistent pattern emerges in terms of the expected number of protein-coding genes. Birds, with their smaller genomes and low number of repeat regions, have approximately 16,000 protein-coding genes, while mammals have closer to 20,000. Fish gene counts are highly variable, ranging between 20,000 and 60,000 protein-coding genes, which is reflective of the multiple rounds of whole genome duplication across fish species.

Non-coding genes, which include pseudogenes, long and short non-coding RNAs, are considerably more variable in terms of overall counts. In particular, lncRNAs annotations are highly variable as they are detected through transcriptomic data and are often expressed at low levels and tissue-specific (Uszczynska-Ratajczak et al., 2018). As a result, species with large amounts of transcriptomic data, sampled across a broad set of tissues and development stages, have considerably more lncRNA annotations than species with a small amount of transcriptomic data. Sequence similarity to species with entries in miRBase (Kozomara et al., 2019) and Rfam (Kalvari et al., 2018) heavily affects annotation of small ncRNAs, species with high sequence similarity to species with good coverage in these databases will generally have higher counts of sncRNAs.

### Comparative Livestock Data

All livestock species are included as part of our protein and ncRNA gene tree pipelines, which calculate gene tress across all species in Ensembl.

Table 2 provides more information on the other comparative data available across different sets of species. These data are calculated for subsets of the species in Ensembl; the selection is made based on the quality of the genome assembly and the clade the species belongs to. For example, the high-quality 57 mammals EPO alignments are clade-specific and require that the genome assemblies used in the alignment are at the chromosome-level, while the 111 eutherian mammals EPO-extended alignment is a broader sampling of mammals and extends to lower quality genome assemblies.

**TABLE 2:** Availability of whole genome alignments and syntenies for livestock and companion animal species.

**Species**	**16 pig breeds**	**57 mammals**	**111 eutherian mammals**	**95 amniota vertebrates**	**36 sauropsids**	**69 sauropsids**	**50 fish**	**86 fish**	**Pairwise**	**Synteny**
	**(EPO-Extended)**	**(EPO)**	**(EPO-Extended)**	**(Mercator-Pecan)**	**(EPO)**	**(EPO-Extended)**	**(EPO)**	**(EPO-Extended)**	**alignments**	**maps**
Chicken				x	x	x			75	24
Duck				x	x	x			2	2
Turkey				x	x	x			2	2
Cat		x	x	x					7	5
Cow	x	x	x	x					26	18
Dog		x	x	x					22	12
Goat		x	x	x					3	3
Horse	x	x	x	x					5	4
Pig	x	x	x	x					19	7
Sheep (texel)	x	x	x	x					6	6
Atlantic herring							x	x	3	2
Atlantic salmon							x	x	3	1
Seabream							x	x	3	2

*X indicates inclusion of a given species in the listed multiple alignments. Methods used to compute multiple alignments are given in brackets. “Pairwise alignments” and “Synteny maps” columns specify the number of available alignments of each type.*

For pig, there are 13 breeds available in Ensembl and we have generated additional breed-specific comparative resources. Table 3 shows data for a 16 species EPO-extended alignment between the 13 pig breeds, in addition to three outgroup species. We have also generated a set of gene trees using these 16 species.

**TABLE 3:** Summary statistics of the pig breeds whole genome alignment.

**Species –**	**Genome len.**	**Genome cov.**	**Genome cov.**	**Coding exon length**	**Coding exon coverage**	**Coding exon coverage**
**breed**	**(gb)**	**(gb)**	**(%)**	**(bp)**	**(bp)**	**(%)**
Pig – Reference	2.50	2.35	93.82	35,828,571	34,187,392	95.42
Pig – USMARC	2.76	2.30	83.49	35,166,034	32,878,028	93.49
Pig – Wuzhishan	2.51	2.29	91.43	32,216,998	30,303,931	94.06
Pig – Tibetan	2.44	2.24	91.88	33,242,148	31,601,348	95.06
Pig – Meishan	2.47	2.29	92.96	34,405,547	33,144,362	96.33
Pig – Jinhua	2.45	2.30	93.54	34,359,345	33,112,715	96.37
Pig – Rongchang	2.46	2.30	93.47	34,688,475	33,474,298	96.5
Pig – Bamei	2.46	2.29	93.26	34,436,299	33,139,773	96.24
Pig – Largewhite	2.46	2.31	93.84	34,666,066	33,395,166	96.33
Pig – Pietrain	2.44	2.30	94.24	34,368,936	33,123,601	96.38
Pig – Berkshire	2.43	2.29	94.17	34,511,197	33,225,283	96.27
Pig – Hampshire	2.44	2.30	94.41	34,573,096	33,284,528	96.27
Pig – Landrace	2.44	2.30	94.1	34,646,469	33,337,432	96.22
Cow	2.72	2.52	92.83	34,983,666	33,272,562	95.11
Horse	2.51	2.28	90.93	37,559,221	34,821,694	92.71
Sheep (texel)	2.62	2.49	95	32,776,750	30,904,741	94.29

*This alignment was initially generated in Ensembl release 98 and is composed of 11,721 blocks (up to 953,990 bp long). Cow, horse, and sheep were used as outgroups in the alignment.*

### Variation Livestock and Companion Animal Resources

Ensembl has variation data for ten livestock and companion animal species, summarised in Table 4. Between 9,000 and 104 million short variants are available for each of these species; in addition, structural variants are available for cow, dog, horse, pig, and sheep. Sources of short variants for livestock and companion animals in Ensembl are dbSNP, Pig SNP Consortium and EVA study PRJEB34225, while all structural variants are imported from DGVa. Sources of the phenotype data are OMIA, GOA and AnimalQTLdb, while allele frequencies are from NextGen Project, International Sheep Genome Consortium and EVA studies PRJEB34225, PRJEB24066, and PRJEB9799. Similar to the variant data, the amount of linked data available for the different species varies significantly. The richest linked data sets with all data types are available for Cow, Dog, Horse, and Sheep, while the linked data sets for Atlantic salmon, Cat and Turkey are most limited.

**TABLE 4:** Variation data counts for livestock and companion animal species.

**Species**	**Short variants**	**Structural variants**	**Populations with allele frequency data/Total number of individuals**	**Variants with**			**Phenotype data**	
				**Population genotypes**	**Sample genotypes**	**SIFT scores**	**Phenotypes**	**Gene associations**
Atlantic salmon	10.1 M	ND	3/80	10.1 M	10.1 M	ND	ND	ND
Cat	3.6 M	ND	ND	ND	ND	7 K	63	64
Chicken	24 M	ND	ND	3.2 M	3.2 M	229 K	225	5 K
Cow	104 M	18 K	1/8	10 K	10 K	2.1 M	549	98 K
Dog	5.9 M	104 K	1/219	727 K	727 K	50 K	257	258
Goat	37 M	ND	5/195	ND	ND	92 K	11	11
Horse	21 M	193 K	1/6	1.1	1.1 M	98 K	88	852
Pig	67 M	224 K	ND	175	15	218 K	394	20 K
Sheep	61 M	2	68/633	147	64	222 K	172	2 K
Turkey	9 K	ND	ND	48	ND	ND	29	42

*The counts are based on Ensembl release 101. Sheep data are from the texel breed. Data sources: Short variants – EVA study PRJEB34225 (Atlantic salmon), dbSNP and Pig SNP Consortium (Pig), dbSNP (others); Structural variants – DGVa (all); Allele frequencies – EVA study PRJEB34225 (Atlantic salmon), NextGen Project (Cow, Goat), EVA study PRJEB24066 (Dog), EVA study PRJEB9799 (Horse), NextGen Project and International Sheep Genome Consortium (Sheep); Phenotypes – OMIA (Cat, Dog, and Goat), GOA and OMIA (Turkey), AnimalQTLdb and OMIA (others). ND, no data.*

For each variant, we also identify all overlapping Ensembl transcripts and provide the most severe consequence of the variant, as defined by sequence ontology^2^. For missense variants of cat, chicken, cow, dog, goat, horse, pig, and sheep, SIFT scores are provided to help assess the potential pathogenicity of the variants.

### Files Available for Livestock Species

Running analyses locally is made easier by the availability of data that has been exported from the Ensembl database into popular file formats for download. Files on the FTP site are organised by releases, with older releases stretching back almost 20 years. As such it is possible to retrieve files representing different reference assemblies or annotations through time. Table 5 shows some of the most popular files available on the FTP site along with a brief description of their content.

**TABLE 5:** Selected files available on the Ensembl FTP site for livestock species.

**Data**	**File types**	**Description**
Genome sequences	FASTA, EMBL, GenBank	The non-redundant genome sequences (sometimes referred to as “toplevel”). Available in softmasked, hardmasked, and unmasked variations
Transcript sequences	FASTA	Sequences for all transcript structures including any 5′ and 3′ UTR present. A cDNA file contains all coding transcript sequences, while non-coding transcripts are represented in a separate ncRNA file
Peptide sequences	FASTA	Amino acid translations for each transcript with an annotated open reading frame
Gene annotation	GFF3, GTF, EMBL, GenBank	Files containing information on genes, transcripts exons and cds structures. The exact content varies from between formats, with GFF3 and GTF being less verbose than EMBL and GenBank formats
Genome alignments	lastZ Net, MAF	Pairwise lastZ genome alignments are available for all species against the reference for their clade. EPO multi-genome alignments are available for various clades including mammals and pig breeds
Variation data	VCF, VEP cache	Variation data is available in VCF format for several species including chicken, cow, goat, pig, and sheep. VEP cache files allow local installation of the VEP to speed up the analysis by using the data stored in the cache

*Files can be found on ftp://ftp.ensembl.org/pub/, with files relating to the current release found in subdirectories prefixed with “current_.” Files from all previous releases back to release 19 are also available.*

## Discussion

Ensembl contains a wealth of livestock data that can be accessed in a number of different ways as described in the protocols presented here. The depth of available data reflects the commercial value of applying genomics to understanding these species and breeds and there is, of course, some variability in the data available for different species. We have certain key data types available for all livestock species including gene annotations, gene trees, inclusion in whole genome alignments and compatibility with tools such as our BLAST service and the Ensembl VEP. In addition to this, there are a significant number of additional data tracks, though this varies from species to species. For example, while all livestock species have associated transcriptomic data used to produce their gene annotations, the amount of transcriptomic data varies considerably. For pig, there is an extensive collection of both short and long read tissue data, all of which are available in the browser as tissue-specific gene tracks. For several species, we have variation data, with tracks for SNPs and structural variants as well as an LD calculation tool available for goat, salmon and sheep. With falling costs of generating these data, we expect that data availability will become more consistent across species. Ensembl will continue to integrate new data in line with the needs of the livestock community.

Working with Ensembl can be divided into two major approaches: working through the genome browser or fetching data and running local analyses. These two approaches are often done in tandem, with analysis starting in the browser, before continuing on to large-scale local analyses using the REST service and/or downloading files from the FTP site.

The browser provides access to many different views of the underlying data. The gene and location views support access to small scale information on a gene or set of genes, while more specialised views such as the synteny view allow for cross species comparisons across genomic regions. A knowledge of the available views is key to utilising the browser fully. The default option for each view tries to strike the right balance between delivering key information without overloading the view with too much visual noise. Additional tracks, that are not visible by default, are accessible from the configuration menu. An example of these are the transcriptomic data tracks, which are available in the gene and location views. For species with transcriptomic data, it is possible to switch on tracks showing the transcripts identified in each tissue/development stage along with intron support and BAM/BigWig coverage plots. These additional tracks can provide valuable extra information for each locus. In addition to understanding available views and tracks, efficiently working with the browser benefits from knowledge of the supported tools. The BLAST/BLAT service can be used to help identify unannotated genes or exons in a genomic region of interest, while the Ensembl VEP can be used to analyse uploaded variants against reference genomes, annotations and linked data. BioMart is a powerful tool for exporting complex data sets. Taking the time to become familiar with the available tools, their strengths and limitations is an important aspect of fully utilising the genome browser.

For large-scale or custom analyses, the path to interacting with Ensembl generally shifts from the browser to programmatic access via the API and bulk download of data via the FTP site. A typical workflow could involve downloading the softmasked genome sequences from the FTP site for a set of species to be analysed, followed by fetching annotation data, such as the gene sets, either from the files on the FTP site or via the REST API. The REST API is a powerful method to subsample or filter the data in various ways, such as only selecting genes on a certain chromosome or fetching a particular subclass of genes such as miRNAs. If no filtering or grouping of the data are required, the corresponding FTP files are generally the most straightforward and fastest way to get bulk access to annotation data. When combined, the FTP site and REST API give access to the vast wealth of data present in Ensembl. They act as a starting point for local workflows and a better understanding of what can be fetched directly from Ensembl can help accelerate downstream analysis.

While the protocols provided here give a comprehensive overview of what data are in Ensembl and how to interact with it, there is still much more to discover. Support is available in a variety of ways including a dedicated email helpdesk (helpdesk@ensembl.org) to field any inquiries about Ensembl. We are currently running virtual training courses during the COVID-19 pandemic, and will resume a full in-person and virtual training programme, including webinars, when possible. Our training materials are accessible online at https://training.ensembl.org. Ensembl courses are also available from the EMBL-EBI Train Online platform^3^. Tutorial videos and recorded webinars can be found both on our YouTube^4^ and Youku^5^ channels.

We invite the community to contact us via our helpdesk to ask questions regarding the use of our browser, tools and related resources, to request training events or to suggest features which would assist their work.

## Data Availability Statement

The original contributions presented in the study are included in the article/supplementary material, further inquiries can be directed to the corresponding author.

## Author Contributions

AG, MS, and FJM designed the protocols. AG, MS, FJM, and PF wrote the manuscript. All authors contributed to the article and approved the submitted version.

## Conflict of Interest

PF is a member of the Scientific Advisory Boards of Fabric Genomics, Inc. and Eagle Genomics, Ltd. The remaining authors declare that the research was conducted in the absence of any commercial or financial relationships that could be construed as a potential conflict of interest.

## Footnotes

1 http://e101.ensembl.org

2 http://www.sequenceontology.org

3 https://www.ebi.ac.uk/training/online/

4 https://www.youtube.com/user/EnsemblHelpdesk

5 https://i.youku.com/i/UMzM1NjkzMTI0?spm=a2hzp.8244740.0.0
